# Comparison of a New Video Intubation Stylet and McGrath® MAC Video Laryngoscope for Intubation in an Airway Manikin with Normal Airway and Cervical Spine Immobilization Scenarios by Novice Personnel: A Randomized Crossover Study

**DOI:** 10.1155/2021/4288367

**Published:** 2021-11-10

**Authors:** Jin-Woo Park, Sungmin An, Seongjoo Park, Francis Sahngun Nahm, Sung-Hee Han, Jin-Hee Kim

**Affiliations:** ^1^Department of Anesthesiology and Pain Medicine, Seoul National University Bundang Hospital, Seongnam 13620, Republic of Korea; ^2^Department of Anesthesiology and Pain Medicine, Seoul National University College of Medicine, Seoul 03080, Republic of Korea

## Abstract

The use of both a video laryngoscope and a video intubation stylet, compared with the use of a direct laryngoscope, is not only easier to learn but also associated with a higher success rate in performing endotracheal intubation for novice users. However, data comparing the two video devices used by novice personnel are rarely found in literature. Nondelayed intubation is an important condition to determine the prognosis in critically ill patients; hence, exploring intubation performance in various situations is of clinical significance. This study is aimed at comparing a video stylet and a video laryngoscope for intubation in an airway manikin with normal airway and cervical spine immobilization scenarios by novice personnel. We compared the performance of intubation by novices between the Aram Video Stylet and the McGrath® MAC video laryngoscope in an airway manikin. Thirty medical doctors with minimal experience of endotracheal intubation attempted intubation on a manikin five times with each device in each setting (normal airway and cervical spine immobilization scenarios). The order of use of the devices in each scenario was randomized for each participant. In the normal airway scenario, the Aram stylet showed a significantly higher rate of successful intubation than the McGrath® (98.7% vs. 92.0%; odds ratio (95% CI): 6.4 (1.4–29.3); *p* = 0.006). The intubation time was shorter using the Aram Stylet than that using the McGrath® video laryngoscope (*p* < 0.001). In the cervical immobilization scenario, successful endotracheal intubation was also more frequent using the Aram stylet than with the McGrath® (96.0% vs. 87.3%; odds ratio (95% CI): 3.5 (1.3–9.0); *p* = 0.007). The Aram Stylet intubation time was shorter (*p* < 0.001). In novice personnel, endotracheal intubation appears to be more successful and faster using the Aram Video Stylet than the McGrath® MAC video laryngoscope.

## 1. Introduction

Video laryngoscope and video intubation stylet have recently become widely accepted devices in airway management as they provide a proper glottic view in patients when conventional direct laryngoscopy is difficult [1, 2]. These video devices have been reported to be easier to learn while contributing to a higher success rate of endotracheal intubation when compared with the conventional Macintosh direct laryngoscope, especially in novice personnel [3–7]. During endotracheal intubation, the video intubation stylet does not require manipulation of the epiglottis to perform glottic exposure, which is necessary when using a conventional or video laryngoscope. Therefore, video intubation stylets seem to be more suitable than video laryngoscopes in patients with limited mouth opening and neck extension [1].

To the best of our knowledge, there are no data comparing the use of the two video devices by novice personnel. Because prompt intubation is very important in emergencies that can occur at any time and under any condition, it should be of great significance to compare intubation performances between the two indirect devices in novices and experts.

We hypothesized that the video stylet would be more successful and faster than the video laryngoscope in endotracheal intubation for novices, since they are not yet skilled in epiglottic manipulation using a laryngoscope blade. It might also be difficult for them to handle a laryngoscope and an endotracheal tube simultaneously since a video intubation stylet is combined within the tube to directly show the tube advance.

Therefore, this study was performed to compare the two video devices, Aram Video Stylet (AVS; Aram Huvis, Seongnam, Korea) and McGrath® MAC (Aircraft Medical Ltd., Edinburgh, UK) video laryngoscope, with respect to the performance and ease of intubation by novice personnel in an airway manikin with normal airway and cervical spine immobilization scenarios.

## 2. Materials and Methods

### 2.1. Study and Participants

The protocol of this study was approved by the Institutional Review Board of Seoul National University Bundang Hospital (research number: B-1905-538-301, 18/04/2019) and was registered prior to participant enrollment in the UMIN Clinical Trials Registry (http://www.umin.ac.jp/english/; registration number: UMIN000036800, 20/05/2019). Written informed consent was obtained from all participants. Medical doctors working at Seoul National University Bundang Hospital with minimal clinical experience of endotracheal intubation were enrolled in this study. Physicians who had performed intubation more than three times, with either a direct or a video device, were excluded from this study.

### 2.2. Airway Devices and Airway Scenarios

**Figure 1:**
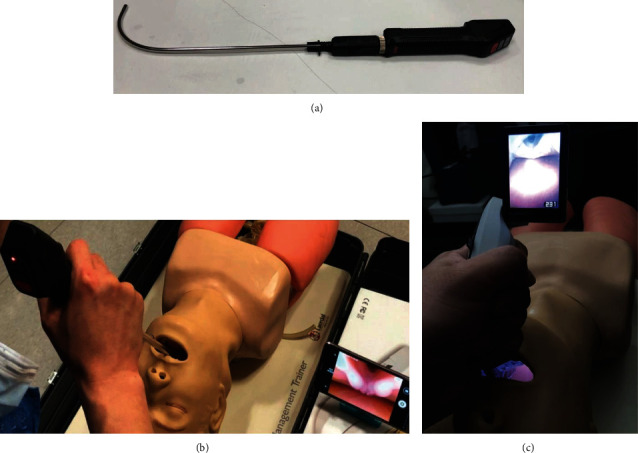
Video devices. (a) AVS consisting of a malleable stylet and handling body. (b) Glottic view from the camera at the end of the stylet of AVS. (c) Glottic view during intubation with McGrath® MAC video laryngoscope. AVS: Aram Video Stylet.

AVS is a newly developed, malleable, hockey stick-shaped video stylet with a rechargeable battery (Figure 1(a)). AVS can be connected to a smartphone or tablet gadget to display the laryngeal view, which aims at reducing the product size and improve its portability. It sends the image data to a mobile device in real time via a wired or wireless connection. The camera is at the end of the stylet, and users can see the image on the mobile display (Figure 1(b)). McGrath® MAC, one of the most popular video laryngoscopes, has a camera at the tip of the blade holding area, and users can see the image through the attached monitor (Figure 1(c)).

For the purpose of this study, there were two airway scenarios: normal airway scenario and cervical spine immobilization scenario. For the normal airway scenario, endotracheal intubation was performed in a Laerdal Airway Management Trainer (Laerdal®, Stavanger, Norway) in the supine position. For the cervical spine immobilization scenario, a rigid neck collar was placed on the manikin to simulate a difficult airway with limited mouth opening and neck mobility.

### 2.3. Endotracheal Intubation Attempts

Before starting the study, each participant underwent a 5-minute tutorial by a single instructor on how to use the two video devices. After the 5-minute tutorial, participants were able to practice using both devices—one attempt per device—under a normal airway scenario with the instructor. Following the lesson, each participant attempted endotracheal intubation five times per device in each airway scenario. The normal airway scenario was attempted first, followed by the cervical spine immobilization scenario.

**Figure 2:**
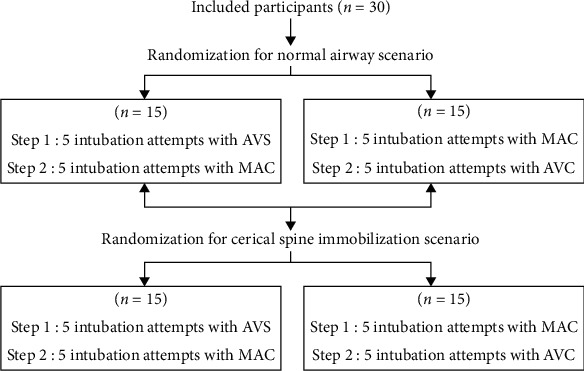
Randomization and intubation trial sequence.

This study was designed as a randomized crossover trial. The randomization list of device sequences in each scenario was generated using a computer-generated code (Random Allocation Software version 1.0; University of Medical Sciences, Isfahan, Iran) with a block size of 4 and a 1 : 1 allocation ratio. The randomization and intubation trials are shown in Figure 2.

During the study, endotracheal intubation was performed with a size 7.0 cuffed endotracheal tube (Mallinckrodt Medical, Athlone, Ireland). In attempts to intubate an endotracheal tube preloaded on the AVS, the stylet was inserted through the mouth and positioned at the glottic opening. The participants confirmed the view from the stylet tip through the display of Galaxy S6 (Samsung, Suwon, Korea) connected to the AVS by a wire (Figure 1(b)). Before the trial of endotracheal intubation with the McGrath® MAC laryngoscope, a malleable stylet was inserted into the endotracheal tube, which was then bent into the same curvature as the blade of the McGrath® laryngoscope; blade #4 was used. For McGrath® intubation, the blade was inserted through the mouth and maneuvered to obtain proper laryngeal exposure on the monitor. In both scenarios with both devices, the endotracheal tube was advanced into the trachea after the tip of the stylet was slightly passed through the vocal cord.

### 2.4. Study Outcomes

The primary outcome was the success rate of endotracheal intubation, which is thought to be clinically important. After each intubation trial was completed, participants connected the endotracheal tube to a self-inflating bag and squeezed it. Successful endotracheal intubation was confirmed with the accomplishment of chest inflation. Failure of intubation was defined as failure of intubation within 60 s or esophageal intubation.

Secondary outcomes included the intubation time, incidence of dental clicks and esophageal malposition, and difficulty level in handling each device. The intubation time was recorded beginning from picking up the video device to the passage of the endotracheal tube through the glottis. In the event of failed intubation, the intubation time of the attempt was noted as 60 s. The incidence of dental clicks due to severe pressure on the teeth of the airway trainer was also recorded. After all intubation trials, each participant was asked to evaluate the difficulty with respect to handling the two video intubation devices based on a numerical rating scale (0, very easy; 10, very difficult).

### 2.5. Sample Size

During a pilot study in which eight novice users performed 40 intubation trials using each device under the normal airway scenario (under the same conditions as in this study), the success rates of endotracheal intubation were 85% and 95% for the McGrath laryngoscope and AVS, respectively. Based on the pilot data, a power analysis was performed using G∗Power 3.1.2 (Heinrich-Heine University, Düsseldorf, Germany). A sample size of about 150 attempts per device was calculated to be required with a power of 0.8 and a significance level of 0.05.

### 2.6. Statistical Analysis

The frequencies of successful intubation, dental click, and esophageal intubation were analyzed using the chi-square or Fisher's exact test, as appropriate. The odds ratio was calculated to determine the probability of AVS presenting each of them, compared to McGrath® MAC. To compare the intubation time and the degree of difficulty regarding device handling between the two intubation devices, Student's *t*-test and Mann–Whitney *U* test were used, respectively. Repeated measures ANOVA was used to test the effect of the attempt number on intubation time (learning curve). For each trial number, the difference in intubation time between the AVS and the McGrath® laryngoscope was analyzed using the Mann–Whitney *U* test.

## 3. Results

Thirty medical doctors participated in this study. None of the patients had prior clinical experience with a video laryngoscope or video stylet. All participants attempted endotracheal intubation five times with each video device per scenario, and all data were used for analysis.

In the normal airway scenario, the AVS showed a significantly higher rate of successful intubation than the McGrath® laryngoscope (98.7% vs. 92.0%; odds ratio (95% CI): 6.4 (1.4–29.3); *p* = 0.006; Table 1). Intubation time was shorter with the AVS than with the McGrath® MAC video laryngoscope (mean (SD); 19.0 (7.7) sec vs. 28.0 (12.8) sec; mean difference (95% CI): 9.1 (6.6–11.5); *p* < 0.001). Dental clicks were less frequent with the AVS than with the McGrath® video laryngoscope (4.7% vs. 25.3%; odds ratio (95% CI): 0.1 (0.1–0.3); *p* < 0.001). The incidence of esophageal malposition was comparable between the two devices (*p* = 0.498).

Similar results were observed in the cervical spine immobilization scenario (Table 2). Successful endotracheal intubation was more frequent with the AVS than with the McGrath® laryngoscope (96.0% vs. 87.3%; odds ratio (95% CI): 3.5 (1.3–9.0); *p* = 0.007). The AVS showed a shorter intubation time (mean (SD): 25.4 (10.2) vs. 35.1 (13.0); mean difference (95% CI): 9.7 (7.1–12.4); *p* < 0.001) and a lower incidence of dental clicks (14.7% vs. 38.7%; odds ratio (95% CI): 0.3 (0.2–0.5); *p* < 0.001). Esophageal malposition occurred comparably between the two video devices (*p* = 0.622).

**Figure 3:**
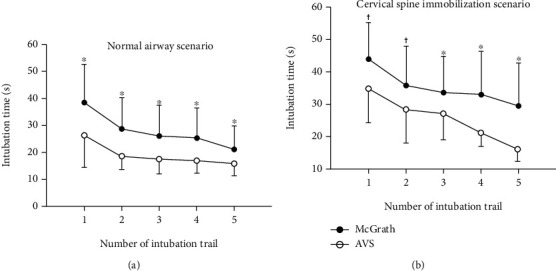
Intubation times with the AVS and McGrath® laryngoscope for every attempt number. ^∗^Significant (*p* < 0.001) difference; ^†^significant (*p* < 0.01) difference between the two devices at each trial number. Error bars are means with standard deviations. AVS: Aram Video Stylet.

In both scenarios, the intubation time of the AVS and McGrath® laryngoscope was reduced with an increasing trial number, suggesting a learning curve (*p* < 0.001 for both devices in both scenarios; Figure 3). The intubation time was significantly shorter with the AVS than with the McGrath® for every attempt number. The learning curve for each video device was not significantly different between the two devices in both airway scenarios (*p* = 0.234 and 0.176 for both scenarios, respectively). The difficulty of device control in novice users was lower for the AVS than for the McGrath® MAC laryngoscope (median (IQR): 4.0 (3.0–5.0) vs. 5.5 (5.0–6.0); *p* < 0.001; Table 3).

## 4. Discussion

In this randomized crossover study, we demonstrated that endotracheal intubation with the AVS resulted in a higher success rate, shorter intubation time, and fewer dental complications in novice personnel, when compared with the McGrath® MAC video laryngoscope.

A key difference between the video intubation stylet and video laryngoscope is that the video laryngoscopy requires manipulation of the larynx tissue to secure the view of the glottis beneath the blade tip, which is a difficult task for novice personnel. On the other hand, the video intubation stylet can directly confirm the glottic view from the tip of the stylet without manipulation. The “hockey-stick” curvature was reported to help endotracheal tube delivery to the glottic opening [8].

While video laryngoscopy may provide a good view of the glottis, it may also be difficult for novices to maneuver a tube to that opening. Because the camera of the video laryngoscope is located in the blade holding area, the viewing direction does not match the advance of an endotracheal tube, making it more difficult for novice users to handle a tube. On the other hand, the video stylet shows the sight from the tip of an endotracheal tube by which users can directly confirm the tube position. Furthermore, the bulky blade of the video laryngoscope may be uncomfortable for novice users to operate and be susceptible to dental contact during intubation [1]. In fact, in our study, novice participants reported that the AVS was less difficult to control than the McGrath® video laryngoscope. In both airway scenarios, the incidence of dental click was also significantly lower during AVS intubation than during McGrath® MAC intubation, with odd ratios of 0.1 and 0.3 for normal and cervical immobilized scenarios, respectively.

Meanwhile, the number of esophageal malposition events was too small to show a statistical difference in our results; a glottic view assisted by the video camera in each device might be helpful to recognize the esophageal malposition of an endotracheal tube. In a previous study, intubation with a video laryngoscope showed significantly fewer cases of esophageal intubation than that with a direct laryngoscope [9].

Endotracheal intubation is a life-saving procedure that provides oxygenation to patients with respiratory failure or loss of airway patency in various clinical situations. Prompt intubation has been reported to improve the prognosis of critically ill and injured patients [10, 11]. However, intubation is a difficult and infrequent procedure for most medical personnel, except for those in specific departments with immense experience, such as anesthesiology or emergency medicine. Furthermore, intubation proficiency may regress if it is not regularly experienced or practiced [3].

Direct laryngoscopy using a Macintosh blade is the conventional method for intubation. However, it has a steep learning curve and remains a difficult skill to master, especially for novice personnel with minimal experience and practice [3, 12–14]. Previous studies have reported that indirect video devices may provide better intubation conditions than direct Macintosh laryngoscopes [3–5, 15–17]. However, the data comparing the two indirect methods in novice users is lacking in literature.

An exact and prompt endotracheal intubation is necessary for emergency situations that do not allow enough time to wait for a physician who is skilled in intubation, and failure to intubate on time can lead to an extremely poor prognosis [18, 19]. The level of operator experience and use of a video laryngoscope compared to a direct laryngoscope are known to be associated with first-attempt intubation success in a general ward setting [20]. Our results suggest that video intubation stylets are more helpful than video laryngoscopes in situations where inexperienced doctors might perform endotracheal intubation. Every attempt to increase the success rate and speed of intubation in various situations is of great clinical value, no matter how small the difference would be. The high portability of the AVS might also be helpful in preparing emergencies that may occur anytime and anywhere.

According to previous studies evaluating various indirect intubation methods, the learning curve of a video laryngoscope or video intubation stylet seems to be more rapid than that of the direct Macintosh laryngoscope [3–5, 7, 21]. In the current study, novices showed a significant learning effect for both video devices within a short intubation experience.

In our intubation protocol with the AVS, an image from the camera was shown on the mobile display connected to the AVS by a wire. The AVS can also send the image data in real time to a mobile device via a Wi-Fi connection. If participants attempted intubation via a Wi-Fi connection, the handling of the device might have been more comfortable. During the intubation trials with both the AVS and the McGrath® video laryngoscope, there was no delay or heterogeneity between the image on the monitor and the actual movement of the devices.

There are some limitations to our study. First, this was a manikin study and did not completely reflect a live clinical condition. Laryngeal compression, which is known to improve glottic exposure during endotracheal intubation, cannot be applied to an airway management trainer. Fogging or contamination of the video device camera by secretion also did not exist in the manikin setting; the condition of intubation was the same for every trial with AVS or McGrath®. However, this study provides additional clues to improve patients' prognosis in clinical situations when novices might perform endotracheal intubation. Further clinical studies are required to confirm our results.

Second, the participants in this study performed intubations with just one product in each type of video intubation device. The generalizability of this study to other devices may be limited.

Third, in our protocol, the AVS was connected to a mobile display via a wired connection. Further studies are needed to determine whether a Wi-Fi connection would result in delayed images and/or display interruptions.

## 5. Conclusions

This randomized crossover manikin study demonstrated that in novice users, intubation using the AVS was more successful and faster than that using the McGrath® MAC video laryngoscope. Our findings should be validated in future clinical trials.

## Figures and Tables

**Table 1:** Success rate of intubation, intubation time, teeth click during intubation, and esophageal malposition, in normal airway scenario (*n* = 150).

	McGrath®	AVS	*p*	Mean difference (95% CI)	Odds ratio (95% CI)
Success	138 (92.0%)	148 (98.7%)	0.006		6.4 (1.4–29.3)
Intubation time (s)^∗^	28.0 (12.8)	19.0 (7.7)	<0.001	9.1 (6.6–11.5)	
Dental click	38 (25.3%)	7 (4.7%)	<0.001		0.1 (0.1–0.3)
Esophageal malposition	2 (1.3%)	0 (0.0%)	0.498		

Results are shown as mean (standard deviation) or number (proportion). The odds ratio was calculated to determine the probability of the AVS to present each outcome, compared to the McGrath® MAC laryngoscope. ^∗^In the event of a failed tracheal intubation, the intubation time of the attempt was noted as 60 s. AVS: Aram Video Stylet.

**Table 2:** Success rate of intubation, intubation time, teeth click during intubation, and esophageal malposition, in cervical spine immobilization scenario (*n* = 150).

	McGrath®	AVS	*p*	Mean difference (95% CI)	Odds ratio (95% CI)
Success	131 (87.3%)	144 (96.0%)	0.007		3.5 (1.3–9.0)
Intubation time (s)^∗^	35.1 (13.0)	25.4 (10.2)	< 0.001	9.7 (7.1–12.4)	
Dental click	58 (38.7%)	22 (14.7%)	< 0.001		0.3 (0.2–0.5)
Esophageal malposition	3 (2.0%)	1 (0.7%)	0.622		0.3 (0.0–3.2)

Results are shown as mean (standard deviation) or number (proportion). The odds ratio was calculated to determine the probability of the AVS to present each outcome, compared to the McGrath® MAC laryngoscope. ^∗^In the event of a failed tracheal intubation, the intubation time of the attempt was noted as 60 s. AVS: Aram Video Stylet.

**Table 3:** Difficulty of device control.

	McGrath®	AVS	*p*
NRS score	5.5 (5.0–6.0)	4.0 (3.0–5.0)	<0.001

Results are shown as median (interquartile range). AVS: Aram Video Stylet; NRS: numerical rating scale.

## Data Availability

The dataset generated and analyzed during the current study is available from the corresponding author upon reasonable request.
